# GPRC6A Mediates Glucose and Amino Acid Homeostasis in Mice

**DOI:** 10.3390/metabo12080740

**Published:** 2022-08-12

**Authors:** Yumin He, Jingyun Su, Hongrui Gao, Jianzhong Li, Zemeng Feng, Yulong Yin

**Affiliations:** 1 Animal Nutrition and Human Health Laboratory, College of Life Sciences, Hunan Normal University, Changsha 410081, China; 2 Hunan Provincial Key Laboratory of Animal Nutritional Physiology and Metabolic Process, Key Laboratory of Agro-Ecological Processes in Subtropical Region, Institute of Subtropical Agriculture, Chinese Academy of Sciences, National Engineering Laboratory for Pollution Control and Waste Utilization in Livestock and Poultry Production, Hunan Provincial Engineering Research Canter for Healthy Livestock and Poultry Production, Scientific Observational and Experimental Station of Animal Nutrition and Feed Science in South-Central, Ministry of Agriculture, Changsha 410125, China; 3 College of Veterinary Medicine, Hunan Agricultural University, Changsha 410128, China

**Keywords:** glucose, amino acids, GPRC6A, mTORC1, autophagy

## Abstract

GPRC6A, an important member of the G-protein-coupled receptor superfamily, has been widely studied in body health maintenance and related diseases. However, it is still controversial whether GPRC6A plays a vital role in glucose homeostasis, and the role of GPRC6A on amino acid homeostasis has not been reported. In this study, GPRC6A was knocked out in C57BL6 mice, and we found that GPRC6A plays an important role in the glucose metabolism, mainly affecting the glucose clearance capacity and gluconeogenesis in mice. GPRC6A plays an important role in maintaining amino acid homeostasis under dietary restrictions, and this may be realized by participating in the regulation of autophagy. Since a large amount of amino acid is lost from urine in aged GPRC6A^−/−^ mice, it is possible that GPRC6A regulates amino acid homeostasis by affecting the integrity of tissue structure. GPRC6A is involved in the regulation of mTORC1 activation but is not necessary for mTORC1 activation under sufficient nutritional supply. In the absence of exogenous amino acids, the loss of GPRC6A induces the GCN2 pathway activation and excessive autophagy of cells, leading to the overactivation of mTORC1, which may be detrimental to body health and cell survival. In summary, this study provides a theoretical and experimental basis for the metabolic process of GPRC6A in body growth and health.

## 1. Introduction

Serum glucose and amino acids (AAs) levels are strictly regulated in life, an altered profile of glucose and AAs implicates various diseases [1,2]. Food intake temporarily increase serum AAs levels, resulting in insulin release and mTORC1 (Mechanistic Target of Rapamycin Complex 1)-dependent protein synthesis in muscle, which enhances AA uptake [3,4,5]. 

Under the condition of insufficient AA intake, such as short-term fasting, mTORC1 activity decreases, and autophagy is induced to release AAs for maintaining the basic AA requirements of the body [6,7,8]. Long-term malnutrition can lead to decreased plasma AAs [9]. AA homeostasis at the cellular level is mainly regulated by the mTORC1 pathway, and GCN2 (Serine/threonine-protein Kinase GCN2)/ATF4 (Activating Transcription Factor 4). When AAs are sufficient, mTORC1 activation consumes large amounts of AAs to regulate the synthesis of proteins, lipids, purines and lysosomes.

When AA levels are insufficient, mTORC1 inactivation induces autophagy and generates AAs to maintain the basic requirements of cells for AAs [10]. While AA deficiency inhibits mTORC1 activation, it also produces a more specific response through the GCN2/ATF4 pathway to deal with AA imbalance [11,12,13].

Extensive studies showed that multiple AA sensors exit and mediate AAs signals upstream of mTORC1 [14,15]. GPRC6A (G-protein-coupled Receptor Class C Group 6 Member A), a member of G-protein-coupled receptors (GPCRs), has 34%, 28% and 24% homology of AAs sequence with CaSR (calcium sensing receptor), T1R1 (Taste Receptor, Type 1, Member 1) and mGluR1 (Glutamate Receptor, Metabotropic 1) [16]. 

In mice, GPRC6A is expressed in the brain, salivary glands, skeletal muscle, heart, lungs, spleen, kidneys, liver, stomach, testis and early embryos [17,18,19]. Immunofluorescence detection revealed that GPRC6A is expressed on the cell membrane [20]. Alkaline AAs (L-Arg, L-Lys and L-Orn), divalent cations and testosterone are efficient activators of GPRC6A [16,21,22]. It is still controversial whether osteocalcin can effectively activate GPRC6A [23,24,25]. In general, GPRC6A plays a major role in the metabolism as an AA receptor on cell membranes.

In the GPRC6a exon II KO mouse model constructed by Quarles, GPRC6A deletion was found to cause severe glucose intolerance and insulin resistance in mice [18]. However, knockout of GPRC6A exon VI in mice showed neither glucose intolerance nor insulin resistance [26]; however GPRC6A mice showed glucose intolerance when subsequently exposed to a high-fat diet [27]. Therefore, scientists considered whether the metabolic phenotype identified by Quarles was co-existent with obesity. Afterward, full locus GPRC6a KO mice were also constructed but failed to resolve this controversy [28,29,30]. Therefore, whether GPRC6A plays an important role in glucose homeostasis has not been determined.

GPRC6A is an important member of the GPCRs family; however, the role of GPRC6A in AA homeostasis has not been reported. As an AAs sensor upstream of mTORC1, few studies have reported on the role of GPRC6A in mTORC1 activation and its relation to autophagy. Thus, we study the role of GPRC6A in glucose and AAs homeostasis. mTORC1 activation and autophagy were also tested to elucidate potential mechanisms.

## 2. Results

### 2.1. Genotyping and Body Weight of GPRC6A^−/−^ Mice

**Figure 1:**
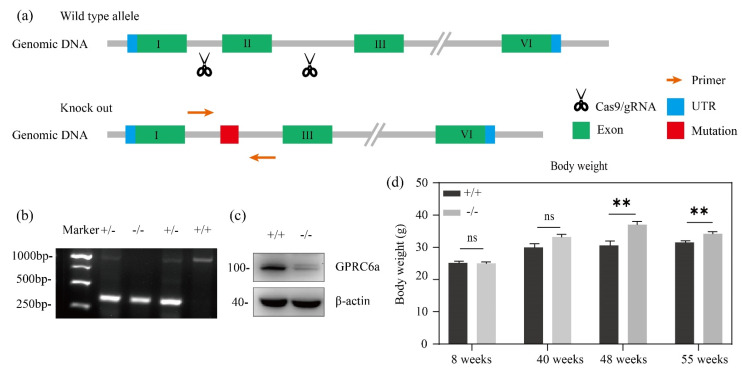
Schematic diagram of gene deletion sites, genotyping and body weight of GPRC6A^−/−^ mice. (**a**) Schematic diagram of gene deletion sites. (**b**) Mice genotyping by PCR and electrophoresis. (**c**) Immunoblotting of protein extracted from mice liver. (**d**) Body weight of GPRC6A^+/+^ and GPRC6A^−/−^ aged 8–55 weeks. ^+/+^ represent WT, ^+/−^ represent heterozygote and ^−/−^ represent KO, the data are shown as the means ± SE. ns, nonsignificant, *p* > 0.05. ** *p* < 0.01, *n* = 8–28.

Mice were genotyped by tissue direct PCR and immunoblotting. As shown in Figure 1b, GPRC6A^+/+^ mice showed only one band on about 1000 bp, and GPRC6A^−/−^ mice showed one band on about 250 bp. This indicated a successful knock out of GPRC6A exon II for 678 bp in this study. These results were further verified by immunoblotting with mice liver (Figure 1c). We fed male GPRC6A^+/+^ and GPRC6A^−/−^ mice a standard chow for rodents from 8 weeks to 55 weeks and found that the body weights of these mice were equal during 8–40 weeks. After being fed for 48 weeks, the body weights of GPRC6A^−/−^ mice were significantly higher than those of GPRC6A^+/+^ mice (Figure 1d).

### 2.2. Serum Glucose Alteration in GPRC6A^−/−^ Mice

**Figure 2:**
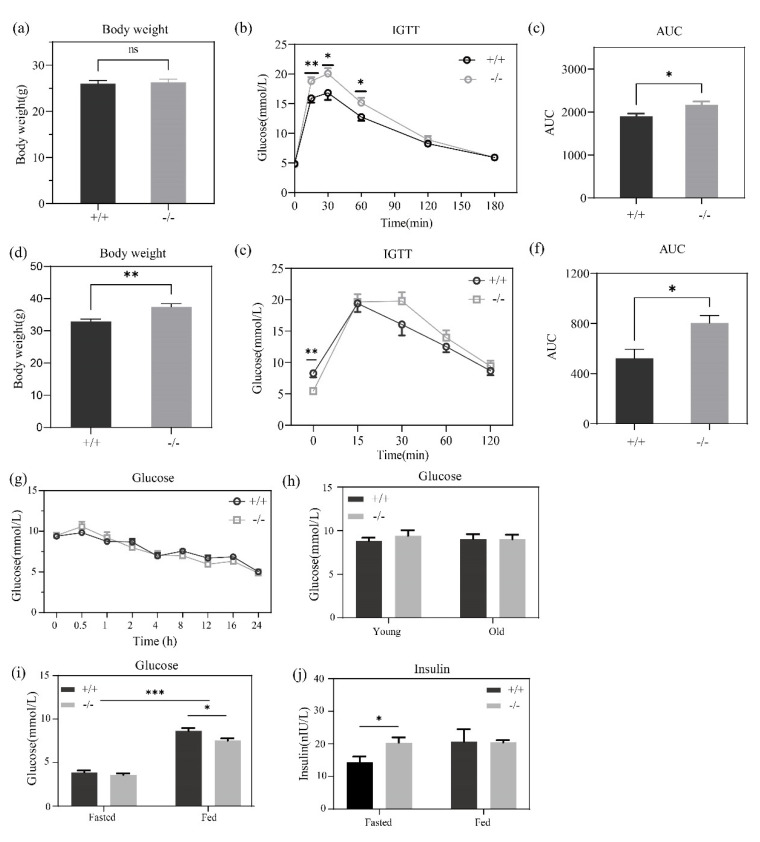
Body weight, IGTT and AUC analysis of mice aged 8 weeks (**a**–**c**) and 54 weeks (**d**–**f**); (**g**) mice serum glucose after 0–24 h fasting; (**h**) serum glucose in young (8 weeks) and old (55 weeks) mice on fed status; (**i**,**j**) serum glucose and insulin of mice fasted for 16 h and after intragastric administration of 2 g/kg lactalbumin powder (aged 8 weeks)**.** The data are shown as the means ± SE. The AUC was calculated independently for every single mouse. ns, nonsignificant. The data were analyzed using an unpaired Student’s *t*-test, *p* > 0.05. * *p* < 0.05, ** *p* < 0.01, *** *p* < 0.001, *n* = 7–10.

We fasted mice aged 8 weeks for 0–24 h, and the blood glucose of these mice was then tested. Interestingly, the results showed that no discrepancy was found during this fasting period, which indicated a similar basal glucose of GPRC6A^+/+^ and GPRC6A^−/−^ mice (Figure 2g). There was also no statistical difference between genotype no matter in young (8 weeks) or old (55 weeks) mice when the mice were fed before glucose testing (Figure 2h). Similar to the results mentioned before, after fasting for 16 h, the serum glucose of GPRC6A^+/+^ and GPRC6A^−/−^ mice aged 8 weeks were identical. However, after the intragastric administration of 2 g/kg lactalbumin powder, glucose in GPRC6A^+/+^ mice recovered faster than GPRC6A^−/−^ mice (Figure 2i), and under the fasted status, higher insulin levels were shown in GPRC6A^−/−^ mice (Figure 2j).

The IGTT (Intraperitoneal Glucose Tolerance Test) was conducted in GPRC6A^+/+^ and GPRC6a^−/−^ mice aged 8 weeks according to a standard protocol [31], these mice were equated in body weight (Figure 2a), and the basal glucose level was found to be identical. However, serum glucose levels were different after intraperitoneal administration of 2 g/kg glucose (Figure 2b). In detail, serum glucose in GPRC6A^−/−^ mice was significantly higher than that of GPRC6A^−/−^ during 15–60 min after glucose administrated, further AUC (Area Under Curve) analysis also showed an obvious discrepancy (*p* < 0.05). 

IGTT was also implemented in mice age 54 weeks. After being fed with a standard chow for rodents for 54 weeks, body weight was significantly higher in GPRC6A^−/−^ mice while basal glucose lever was lower (Figure 2d,e). After weight-matched glucose was intraperitoneal injected, no obvious difference was found during 15–120 min between these mice, AUC of GPRC6A^−/−^ was significantly higher. These results demonstrate an impaired glucose homeostasis of GPRC6A^−/−^ mice.

### 2.3. Impaired Amino Acid Homeostasis in GPRC6A^−/−^ Mice

Blood was collected from 8-week-old mice after 16 h fasting, and the serum AAs were measured after serum separation. As shown in Table 1, we found that, after 16 h fasting, serum levels of His, Gln, Val, Ile and Leu in GPRC6A KO mice were significantly higher than those in WT mice. However, the serum Trp level in KO mice was significantly lower than in the WT group.

We further tested the response to lactalbumin powder after 16 h fasting. Mice aged 8 weeks were fed with 2 mg/kg lactalbumin protein powder after 16 h of fasting, the AA levels of the two groups changed significantly. In KO mice, no AA levels were higher than WT mice, while the contents of Gly, Glu and Cys in serum of WT mice were significantly higher than those of KO mice.

There were no significant differences in the levels of AAs in the 8-week-old mice (Table 2), indicating that, under normal feeding conditions without other external stimuli, GPRC6A deletion had no significant effect on serum AA levels in mice. In the serum of old (55 weeks) mice, the contents of His, Ala and Met in WT mice were significantly higher than those in the KO group without fasting.

**Figure 3:**
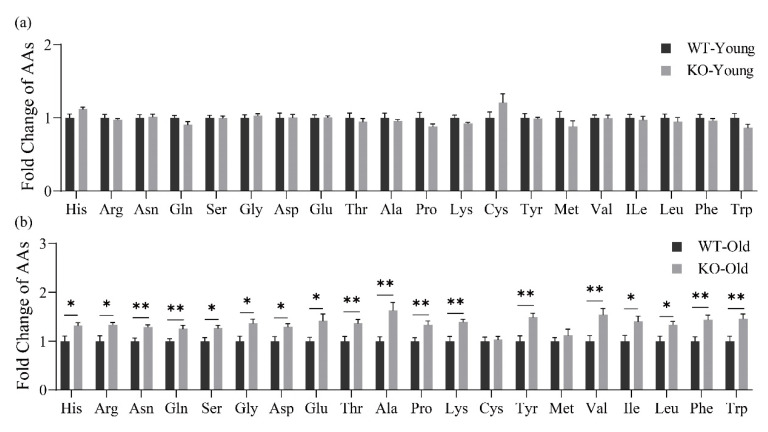
Effects of GPRC6A deletion on free amino acids in the urine of mice at different growth stages, (**a**) (8 weeks), (**b**) (55 weeks). All data were normalized by the average; error bars represent the standard error of the mean. The data were analyzed using an unpaired Student’s *t*-test, * *p* < 0.05, ** *p* < 0.01, *n* = 7–11.

We collected urine from mice aged 8 weeks to test free AAs under the condition of not fasting, results showed that urine free AA levels in KO mice had no significant difference between the WT mice. When mice were fed to 55 weeks, we found that, except for Cys and Met, the levels of other AAs in the urine of KO mice were significantly higher than those of WT mice (Figure 3).

### 2.4. GPRC6a Is Involved in the Regulation of mTORC1 Activation and Autophagy

**Figure 4:**
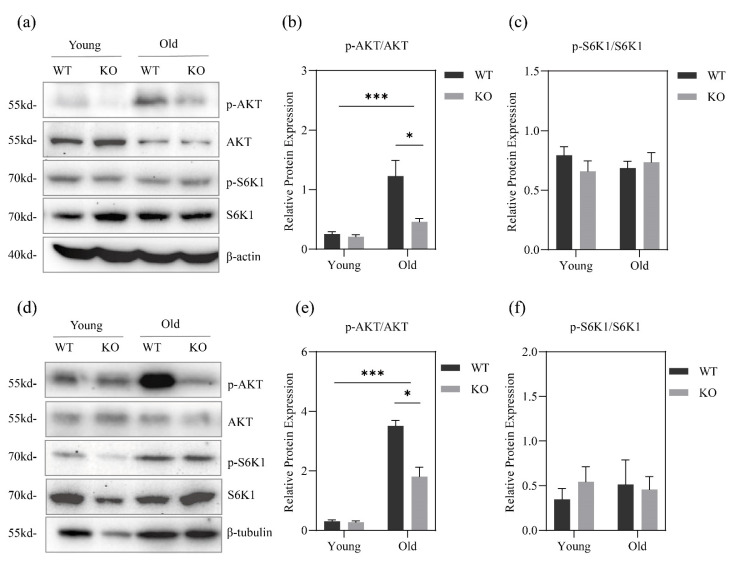
Effects of GPRC6A deletion on the expression of mTORC1 pathway-related proteins in 8-week-old and 55-week-old mice. (**a**) Western blots of mice liver protein expression. (**b**,**c**) Statistics of mice liver protein expression. (**d**) Western blots of mice skeletal muscle protein expression. (**e**,**f**) Statistics of mice skeletal muscle protein expression. Phosphorylated proteins were referenced to total proteins. The data were analyzed by two-way ANOVA and unpaired Student’s *t*-test under the same age, * *p* < 0.05, ***p < 0.001, *n* = 3.

The loss of GPRC6A did not significantly affect the mTORC1 activation (phosphorylation of S6K1) in the liver and skeletal muscle of mice aged 8 weeks and 55 weeks under normal feeding conditions (Figure 4a,c,d,f). Similarly, there was no significant difference in the phosphorylation of AKT in 8-week-old mice (Figure 4a,b,d,e). Age had a significant effect on the phosphorylation level of AKT. The phosphorylation level of AKT in the tissues of mice aged 55 weeks was significantly higher than that of mice aged 8 weeks, and at 55 weeks, the phosphorylation level of AKT in liver and muscle of GPRC6A WT mice was significantly higher than that of KO mice (Figure 4a,c,d,e).

**Figure 5:**
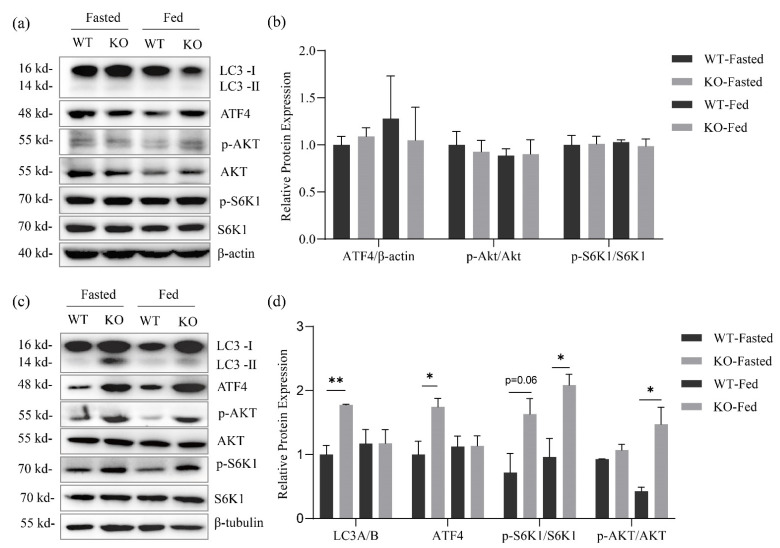
Effects of GPRC6A on tissue protein expression in 8-week-old mice under fasting and re-feeding condition. (**a**) Western blots of mice liver protein expression. (**b**) Statistics of mice liver protein expression. (**c**) Western blots of mice skeletal muscle protein expression. (**d**) Statistics of mice skeletal muscle protein expression. Phosphorylated proteins were referenced to total proteins, non-phosphorylated proteins were referenced to β-actin or β-tubulin. The data were analyzed by two-way ANOVA and unpaired Student’s *t*-test under the same feeding state, * *p* < 0.05, ** *p* < 0.01, *n* = 3.

As shown in Figure 5, the feeding status had no significant effect on the mTORC1 activation level, AKT phosphorylation level, ATF4 and expression of autophagosome formation marker LC3-I/II in liver of 8-week-old mice (Figure 5a,b). The expression levels of ATF4 and LC3-I/II in skeletal muscle of GPRC6A KO mice were significantly higher than those of WT mice under fasting conditions. In addition, although the difference was not significant (*p* = 0.06), the activation level of mTORC1 in KO mice was higher. The activation level of mTORC1 in GPRC6A KO mice was still higher than that in WT mice, and the phosphorylation level of AKT was also significantly higher than that in KO mice (Figure 5c,d).

## 3. Discussion

It has long been controversial whether GPRC6A affects the resting blood glucose in mice. In the first exon II knockout mice, after overnight fasting at 16 weeks of age, the blood glucose of GPRC6A KO mice was significantly higher than that of WT mice [18], and the resting blood glucose of 10-week-old liver organ specific knockout mice was also higher than that of WT mice [30]. In the 17–19-week-old GPRC6A exon VI KO mice constructed by another team, KO and WT mice showed similar resting blood glucose [26], and GPRC6A KO and WT mice also showed similar resting blood glucose values in GPRC6A full-gene knockout mouse models aged 4 weeks and 6–8 weeks [32,33]. 

As some studies in the reported literature did not report the specific duration after fasting that can be considered as resting state, we set multiple time points to measure the blood glucose within 0–24 h to observe whether the difference in resting blood glucose between GPRC6A KO mice and WT mice in the literature was caused by the difference in fasting time. We found that GPRC6A loss had little effect on resting blood glucose in 8-week-old mice, at least for a set period of time (0–24 h). For 8-week-old mice, there was no significant difference in blood glucose between WT and KO mice after fasting for 0 and 16 h.

Therefore, we proved that, in young mice, fasting duration and GPRC6A deletion had no significant effect on resting blood glucose. However, for 12-month-old mice, the glucose level of WT mice was significantly higher than that of KO mice after 16 h of fasting, even though glucose levels of the two genotypes were similar in the non-fasting state, suggesting that some other factors, such as age, may be important in affecting the resting glucose level of mice.

In order to further study the effect of GPRC6A deletion on the glucose clearance ability in mice, IGTT tests were performed in mice aged 8 weeks and 12 months, and we found that GPRC6A deletion affected the glucose clearance ability in male mice aged 8 weeks and 12 months. This is consistent with the previously reported GPRC6A exon II knockout mice and liver organ-specific GPRC6A gene knockout mice [18,30] as well as the results of GPRC6A exon VI KO mice fed with a high-fat diet for 18 weeks [27]. 

When mice were fed with whey lactalbumin powder after fasting for 16 h, the blood glucose recovery rates of WT mice were significantly faster than KO mice. During this process, the rise of glucose level was mainly due to AA gluconeogenesis [7]. Formal research suggested that GPRC6A is related to the process of gluconeogenesis [30]; thus, we further confirmed that GPRC6A was closely related to glucose homeostasis in mice. The plasma insulin level plays an important role in glucose homeostasis. After eating, the rapid increase of glucose level in the body will promote the secretion of insulin to control blood glucose.

Abnormal increases of the insulin level may have adverse effects on the body, such as the excessive accumulation of fat and eventually obesity [34]. In general, the loss of GPRC6A affects the metabolic homeostasis of mice to some extent. Phenotypic differences of IGTT in different GPRC6A knockout mice may be due to the GPRC6A knockout model used. On the other hand, even if the same gene knockout model of the same strain is used, differences in hybridization algebra and mouse age may also cause phenotypic differences of mice. Previous studies have shown that different knockout models of the same gene may also produce different phenotypes [35,36,37].

AA levels, especially in the blood, remain relatively stable, even under dietary protein restriction or fasting status, short-term fasting does not change the level of AAs in the blood, and even long-term fasting only brings about mild changes [38,39]. As an AA receptor on cell membrane, the function of GPRC6A in the maintenance of AA homeostasis has not been reported. In this study, for mice with different genotypes, there are extremely significant differences, and thus GPRC6A plays a significant role in the homeostasis of AAs in the body.

In detail, after fasting for 16 h, the serum levels of His, Gln, Val, Ile and Leu in KO mice were significantly higher than those in WT mice. Interestingly, this is consistent with the AAs accumulated in the lysosomes of SLC38A9-deficient cells after autophagy [8,40]. In fact, under starvation, the body partially generates AAs through autophagy to maintain the body’s AA homeostasis [7,41]. In view of the huge differences in the AAs mentioned above, we hypothesized that GPRC6A may be involved in autophagy to regulate AA homeostasis.

Muscle is the largest AA pool in mammals [42]. We detected the autophagosome formation marker LC3-I/II in skeletal muscle and found that functional loss of GPRC6A led to a higher autophagy level in skeletal muscle of mice, and the expression level of ATF4, a key transcription factor in the GCN2 pathway, was also higher. Therefore, GPRC6a deletion in mice may lead to overactivation of the GCN2 pathway and greater autophagy [11], thereby, activating mTORC1 through free AAs produced by autophagy; this phenomenon was reported before [43].

Studies have shown that autophagy is more likely to occur in the liver than in skeletal muscle under fasting in mice [7]. In this study, feeding status and GPRC6a deletion did not significantly affect mTORC1 activation and the autophagy level in liver of mice. Liver is the central organs of body; therefore, we suspect that, when nutrients were limited, other tissues of the body especially skeletal muscle may produce large amounts of AAs by autophagy to maintain the body’s steady-state.

After feeding the mice after 8 h fasting, results showed that the recovery speed of serum AAs levels in KO mice significantly lags behind that of WT mice. In detail, Gly, Glu and Cys levels were significantly lower than that of WT mice. These results suggest that the lack of GPRC6A not only affects AAs homeostasis under dietary restrictions but also the response speed to dietary protein and AA. Protein ingested by the body generated AAs, partly transported into the urine, and most of them could be reabsorbed back into the blood circulation by the renal tubular [44,45]. 

In order to study whether the lack of GPRC6A impacted reabsorption, we collected urine to determine free AA. At the age of 8 weeks, there was no difference in the AA levels in urine of mice. At the age of 55 weeks, almost all AA levels in urine of KO mice were significantly higher than that of WT mice, indicating that serious AA loss occurred in elderly GPRC6a KO mice, suggesting that, at the age of 55 weeks, GPRC6A deficient mice may have developed severe kidney damage [46,47]. In AA transporter SLC16A9 KO mice, the urine level of free AAs was significantly higher than WT mice; therefore, the loss of tissue AA receptors and transporters may lead to weakened renal reabsorption and thus lead to massive loss of AAs [48].

In summary, this study proved that GPRC6A plays an important role in glucose and AAs homeostasis in mice, GPRC6A acts as an AA receptor upstream of mTORC1, participating in the regulation of mTORC1 activation and autophagy to maintain normal growth and development of the body.

## 4. Materials and Methods

### 4.1. Animals and Diets

Male GPRC6A^−/−^ C57BL/6 mice were obtained from Shanghai Model Organisms Center (Shanghai, China) and backcrossed to WT C57BL/6 mice for at least six generations, GPRC6A^−/−^ and GPRC6A^+/+^ littermate used in formal experiments were generated by heterozygote parents to eliminate genetic backgrounds. All animal procedures were approved by the Animal Ethics Committee of the Institute of Subtropical Agriculture, Chinese Academy of Sciences. Mice were fed with a standard chow for rodents from 8 to 55 weeks of age and were housed at 23 ± 2 °C, 55 ± 5% humidity with 12:12 h light dark cycle and with food and demineralized water *ad libitum*.

### 4.2. Tissue Direct PCR

A Tissue Direct PCR kit (Thermo, F170S, Waltham, MA, USA) was used for genotyping according to the instructions. Mice tails were collected and digested as amplification template, PCR and agarose gel electrophoresis was subsequently performed according to the protocols. Specific primers for GPRC6A are followed: forward primer: AAGGACATGGGGGTTGAGTGAG, reverse primer: AATGACAGATTCCGAGTC CAGC (Sangon Biotech, Shanghai, China).

### 4.3. Immunoblotting

Protein was extracted by ice cold RIPA lysis buffer (Beyotime Biotechnology, P0013B, Shanghai). The lysis was centrifuged at 12,000 rpm for 10 min (4 °C), and the protein concentration in supernatant was quantified using a BCA kit (Beyotime Biotechnology, P0010, Shanghai, China). For all samples, protein with equal concentration (5 μg/μL) was denatured with loading buffer by boiled in hot water for 10 min then loaded on 10% or 15% gels (Servicebio, G0102-200G, Wuhan, China). Proteins in gels were electronically transferred to a Polyvinylidene Fluoride (PVDF) membrane (Millipore, 0.22 or 0.45 μm, Darmstadt, IN, USA) and blocked for 15 min at room temperature by blocking buffer purchased from Beyotime Biotechnology. 

Membranes were incubated with primary antibody overnight (10–16 h) at 4 °C. The primary antibody used were listed as follows: mouse anti-actin antibody, 1:500 (sc-8432, Santa Cruz, Dallas, TX, USA), mouse anti-p70 S6 kinase α antibody, 1:500 (sc-8418, Santa Cruz, Dallas, TX, USA), mouse anti-p-p70 S6 kinase α antibody, 1:500 (sc-8416, Santa Cruz, Dallas, TX, USA), mouse anti-Akt1 antibody, 1:500 (sc-5298, Santa Cruz, Dallas, TX, USA), mouse anti-p-Akt1/2/3 antibody, 1:500 (sc-377556, Santa Cruz, Dallas, TX, USA), mouse anti-CREB-2/ATF-4 antibody, 1:500 (sc-390063, Santa Cruz, Dallas, TX, USA) and rabbit anti-LC3A/B (D3U4C) XP^®^ antibody, 1:1000 (#12741, CST, Boston, MA, USA). 

After being incubated with primary antibody, the membranes were washed by PBST for 10 min (four times). Then, the membranes were incubated at room temperature for 1–2 h with HRP-conjugated goat anti-rabbit or anti-mouse secondary antibodies (Santa Cruz, 1:5000, Dallas, TX, USA) diluted in PBST and then washed by PBST at room temperature for 10 min (four times). The membranes were lastly exposed to electrochemiluminescence, and the pictures were saved for further data analysis.

### 4.4. Intraperitoneal Glucose Tolerance Test

An ACCU-CHEK glucometer (Roche Diagnostics, Shanghai, China) was used to test the plasma glucose. An intraperitoneal glucose tolerance test (IGTT) was performed on GPRC6A^−/−^ and GPRC6A^+/+^ mice aged 8 weeks and 12 months according to a published experimental method [31]. Briefly: (1) At 17:00 pm on the first day, WT and KO mice were weighed and then deprived of feed while having access to water. (2) At 9:00 am the next day, tail blood of the mice was collected to measure glucose, which was recorded as the blood glucose as 0 min. (3) The abdomen of mice was sterilized with 70% ethanol, followed by intraperitoneal glucose (Sigma-Aldrich, D9434, Saint Louis, USA) injection (2 mg/kg body weight). (4) Blood glucose was measured at 15, 30, 60, 120 and 180 min. (5) The mice were placed back into the cage and fed with feed.

### 4.5. Biochemical Assays and Free AAs Quantification

When the experiments were terminated, blood was collected and centrifuged at 3000 rpm for 10 min, the plasma glucose (Roche Diagnostics, Basel, Switzerland) was measured using a commercial kit, and the serum insulin (CUSABIO, CSB-E05071m, Wuhan, China) was evaluated using ELISA. Free AAs in plasma were quantified using an Applied Biosystems 3200 Q TRAP LC/MS/MS system equipped with an RP-C18-column (150 mm length, 4.6 mm diameter and 5 mm particle size) as previously described [49].

### 4.6. Statistical Analysis

Data were analyzed by unpaired Student’s *t*-test or two-way ANOVA using Prism GraphPad Software (9.0) and Statistical Product and Service Solutions (25), data are presented as the means ± SE, statistical significance was set at *p* < 0.05.

## Figures and Tables

**Table 1:** Effects of GPRC6A deletion on serum AAs in mice under fasting and refeeding conditions.

AAs(μM)	WT-Fasted	KO-Fasted	WT-Fed	KO-Fed	SEM	*p*-Value		
						Genotype	Condition	Interaction
His	48.21	56.44 **	49.2	56.65	1.35	0.003	0.805	0.873
Arg	112.2	122.77	85.59	86.86	3.87	0.26	<0.001	0.374
Asn	103.39	107.39	98.47	105.04	2.37	0.286	0.461	0.793
Gln	331.63	472.17 ***	392.06	414.74	11.72	<0.001	0.921	0.001
Ser	79.61	82.35	81.61	79.26	1.72	0.956	0.882	0.49
Gly	253.75	236.28	255.72	230.62 **	4.06	0.009	0.806	0.613
Asp	18.13	18.46	12.61	11.99	0.72	0.877	<0.001	0.619
Glu	81.49	72.13	72.22	58.9 **	2.08	0.0011	0.0012	0.532
Thr	132.99	133.36	139.38	148.88	3.66	0.506	0.146	0.538
Ala	277.06	270.34	278.29	277.91	7.18	0.816	0.774	0.836
Pro	74.78	77.88	77.9	73.16	1.43	0.781	0.787	0.194
Lys	221.72	252.2	238.43	222.45	5.6	0.505	0.549	0.04
Cys	3.42	4.29	11.28	6.67 *	0.69	0.041	<0.001	0.004
Tyr	59.53	54.57	47.6	45.06	1.38	0.056	<0.001	0.526
Met	47.74	49.57	46.69	48.22	0.91	0.383	0.532	0.938
Val	190.45	224.68 **	190.25	206.67	4.46	0.003	0.242	0.253
Ile	81.67	110.19 **	83.41	86.38	3.02	0.001	0.019	0.008
Leu	140.64	185.69 **	143.1	153.34	5.04	0.002	0.07	0.037
Phe	70.07	76.32	71.36	75.09	1.16	0.035	0.9896	0.578
Trp	66.48	57.08 **	84.04	73.08	2.55	0.0104	<0.001	0.833

The data were analyzed using two-way ANOVA and unpaired Student’s *t*-test under the feeding state, * *p* < 0.05, ** *p* < 0.01, *** *p* < 0.001, *n* = 7–11.

**Table 2:** Effects of GPRC6A deletion on serum free amino acids in mice at different growth stages.

AAs(μM)	WT-Young	KO-Young	WT-Old	KO-Old	SEM	*p*-Value		
						Genotype	Age	Interaction
His	49.91	55.85	61.06	51.53 *	1.33	0.444	0.15	0.002
Arg	150.67	146.98	131.63	118.72	4.46	0.309	0.006	0.57
Asn	35.19	35.68	46.68	41.5	1.38	0.325	0.001	0.236
Gln	416.48	377.06	415.65	395.73	8.09	0.072	0.579	0.545
Ser	89.62	89.52	104.73	92.46	2.25	0.146	0.038	0.152
Gly	227.65	234.07	227.43	222.55	4.02	0.926	0.485	0.502
Asp	21.93	22.01	17.84	14.48	0.76	0.152	<0.001	0.133
Glu	105.96	106.7	72.49	70.38	3.46	0.829	<0.001	0.653
Thr	130.33	123.53	139.78	131.37	3.72	0.32	0.259	0.915
Ala	360.19	345.03	352.01	284.52 *	10.45	0.036	0.07	0.175
Pro	109.99	96.82	105.86	85.78	3.76	0.026	0.295	0.631
Lys	343.47	317.29	278.44	250.14	9.22	0.058	<0.001	0.939
Cys	1.19	1.44	1.02	1.09	0.09	0.432	0.21	0.661
Tyr	67.07	66.24	68.42	75.99	1.76	0.331	0.114	0.227
Met	72.01	63.64	94.78	65.38 *	4.2	0.019	0.118	0.177
Val	164.18	163.27	174.03	184.09	4.93	0.647	0.132	0.584
Ile	63.97	62.32	69.33	67.93	1.78	0.679	0.143	0.973
Leu	113.6	108	118.62	112.74	2.9	0.344	0.42	0.982
Phe	66.51	63.96	71.83	69.73	1.4	0.402	0.051	0.935
Trp	88.52	76.52	80.12	95.56	3.02	0.766	0.362	0.023

The data were analyzed by two-way ANOVA and unpaired Student’s *t*-test under the same age, * *p* < 0.05, *n* = 7–11.

## Data Availability

The data presented in this study are available on request from the corresponding author.
